# Cardiac Arrest During a Ferric Derisomaltose Infusion Followed by Complete Heart Block: A Case Report

**DOI:** 10.5811/cpcem.1650

**Published:** 2024-04-17

**Authors:** Michael Scott, Natalie Jansen, Leslie A. Bilello

**Affiliations:** Beth Israel Deaconess Medical Center, Department of Emergency Medicine, Boston, Massachusetts

**Keywords:** *ferric compounds*, *iron*, *heart block*, *case report*

## Abstract

**Introduction:**

Ferric derisomaltose is the newest available parenteral iron formulation. Studies have demonstrated a good safety profile with improved tolerability compared to alternative parenteral iron formulations. To date there have been no reported acute, life-threatening cardiac events associated with ferric derisomaltose.

**Case Report:**

An 86-year-old male who had previously tolerated routine iron infusions received a first dose of ferric derisomaltose at an outpatient infusion clinic. Six minutes into the infusion the patient became unresponsive with no palpable pulse. Return of spontaneous circulation was achieved after two minutes of chest compressions. Electrocardiogram showed complete heart block requiring transcutaneous pacing and vasopressor administration. The patient was transferred to the emergency department for stabilization and then admitted to the cardiac intensive care unit. During admission, the patient received a dual-chamber, permanent pacemaker without complication and was ultimately discharged.

**Conclusion:**

It may be reasonable to consider parenteral iron as a toxicological etiology for patients presenting with complete heart block temporally associated with parenteral iron administration, particularly in patients with underlying conduction abnormalities.

CPC-EM Population Health Research Capsule What do we already know about this clinical entity?

*Ferric derisomaltose is the most recently approved parenteral iron product by the US Food and Drug Administration.*

What makes this presentation of disease reportable?

*We report a case of complete heart block that may have been precipitated by parenteral iron therapy.*

What is the major learning point?

*Although well-tolerated, newer parenteral iron formulations may pose risks that have not yet been elucidated.*

How might this improve emergency medicine practice?

*It may be reasonable for emergency physicians to consider iron chelation therapy if parenteral iron is the suspected etiologic agent of complete heart block.*

## Introduction

Parenteral iron is frequently utilized to treat iron deficiency anemia. A variety of parenteral iron products are available; the newest product in the United States is ferric derisomaltose (FDI) following US Food and Drug Administration approval in January 2020. Ferric derisomaltose was introduced in Europe in 2010 under the name iron isomaltoside. While serious adverse events caused by parenteral iron infusions are rare and typically characterized as anaphylactic-type reactions, FDI allows for high-dose rapid iron infusion with improved tolerability compared to older formulations. Ferric derisomaltose can provide full iron repletion with a single dose, thus reducing the number of infusions required.^1^^,^^2^ Here we describe a case of cardiac arrest six minutes after initiation of FDI, with no obvious signs of anaphylaxis, followed by persistent complete heart block upon achieving return of spontaneous circulation (ROSC).

## Case Report

An 86-year-old, Farsi-speaking male with a past medical history of type 2 diabetes, chronic kidney disease stage IIIb (baseline serum creatinine 1.7–2.2 milligrams per deciliter (mg/dL), iron deficiency anemia, gastroesophageal reflux disease, peptic ulcer disease, colon cancer status post resection, coronary artery disease status post percutaneous coronary intervention in 2016, and Parkinson disease presented to an outpatient infusion clinic for a routine iron infusion. He had previously received ferumoxytol infusions twice monthly with no documented reactions and was being switched to FDI to decrease infusion requirements. Home medications included atorvastatin, calcitriol, carbidopa-levodopa, furosemide, lisinopril, tamsulosin, aspirin, and sodium bicarbonate. He had no known allergies.

**Image 1.**
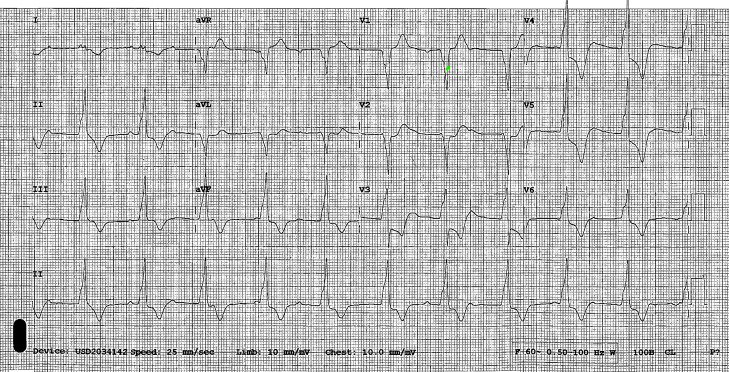
Electrocardiogram showing complete heart block with ST depressions in precordial leads upon initial emergency department presentation at 11:14 am.

Ferric derisomaltose was initiated at 10:10 am. At 10:16 am the patient became unresponsive with agonal breathing and no palpable pulse. Chest compressions were initiated, and ROSC was achieved after one two-minute round of compressions. Epinephrine was not administered. Post-ROSC blood pressure (BP) was 60/30 millimeters of mercury (mm Hg), and point-of-care blood glucose was 220 mg/dL (reference range during fasted state: 70–100 mg/dL). A normal saline 1,000 mL bolus was initiated. The patient was placed on 100% oxygen via nonrebreather mask (NRB); intubation was not required. The patient was transferred to a nearby emergency department (ED) and found to be in complete heart block with significant ST depressions in the precordial leads (Image 1). Transcutaneous pacing was initiated. Heart rate at the time of transcutaneous pacing initiation was not reported. Norepinephrine and vasopressin were initiated following the placement of right tibial intraosseous and right femoral central lines. Pacing was discontinued after a heart rate above the pacing threshold was achieved (60 beats per minute [bpm]). Vasopressin was discontinued, and the patient was transferred to our tertiary-care ED by emergency medical services.

**Image 2.**
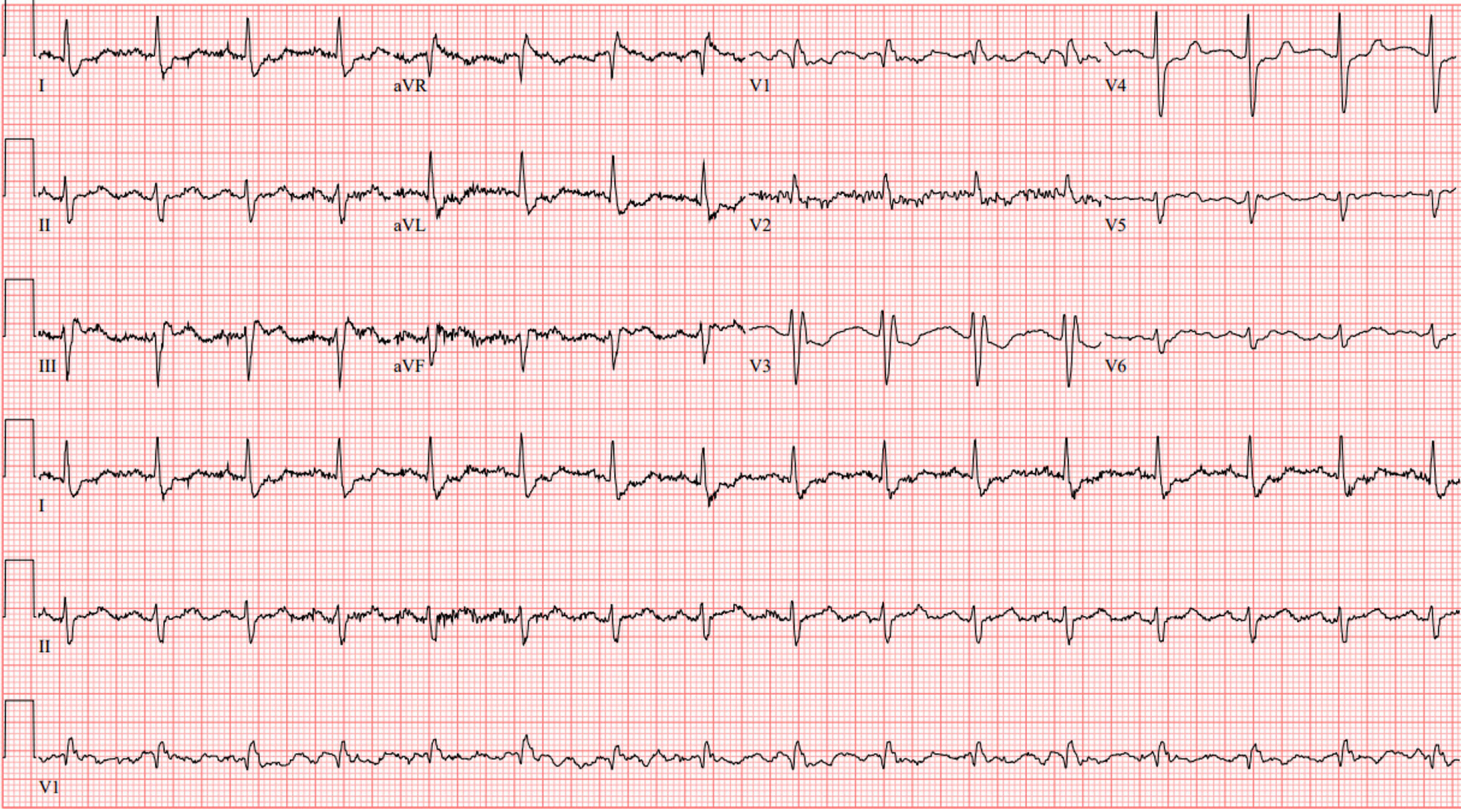
Electrocardiogram upon arrival to tertiary-care emergency department at 1:26 pm.

Upon arrival, the patient’s vital signs were documented as heart rate 92 bpm, BP 136/49 mm Hg, and oxygen saturation 92% on NRB with respiratory rate range 12–37 breaths per minute. Norepinephrine was the only medication infusing for hemodynamic support and was continued at 0.25 micrograms per kilogram per minute. Calcium gluconate 1 gram intravenous (IV) and magnesium sulfate 2 grams IV were administered empirically. On exam, the patient was alert and oriented and exhibited bilateral lower extremity edema. Upon arrival to our ED, electrocardiogram (ECG) was repeated with resolution of previously appreciated ST depressions (Image 2). Therefore, a posterior ECG to investigate for posterolateral myocardial infarction was not performed. Troponin T was slightly elevated at 0.11 nanograms (ng) per mL (reference range 0–0.01 ng/mL) in the setting of chronic kidney disease. Creatinine kinase-MB isoenzyme resulted within normal limits (Table), making acute coronary syndrome less likely. No further acute interventions were required in the ED.

**Table.** Lab results for elderly patient who suffered cardiac arrest following a high-dose rapid iron infusion.

Laboratory test	Result	Reference range
Thyroid stimulating hormone	2.2 μIU/mL	0.27–4.2 μIU/mL
Lyme VsIE/PepC10	Positive	Negative
Lyme disease antibody	Negative	Negative
Iron	33 μg/dL	45–160 μg/dL
Total iron binding capacity	243 μg/dL	260–470 μg/dL
Ferritin	68 ng/mL	30–400 ng/mL
Transferrin	187 mg/dL	200–360 mg/dL
Potassium	4.5 mEq/L	3.5–5.4 mEq/L
Calcium	8.2 mg/dL	8.4–10.3 mg/dL
Magnesium	1.5 mg/dL	1.6–2.6 mg/dL
Phosphorous	3.1 mg/dL	2.7–4.5 mg/dL
Troponin T	0.11 ng/mL	< 0.10 ng/mL
Creatinine	1.9 mg/dL	0.5–1.2 mg/dL
CK-MB	6 ng/mL	0–10 ng/mL

*μIU*, micro-international unit; *mL*, milliliter; *VsIE/PepC10*, *Borrelia burgdorferi* antigens; *μg*, microgram; *dL*, deciliter; *ng*, nanogram; *mg*, milligram; *mEq*, milliequivalent; *L*, liter; *CK-MB*, creatinine kinase-myoglobin binding.

The patient was admitted to the cardiac intensive care unit (ICU), and vital signs at time of transfer from the ED to the cardiac ICU were documented as heart rate 75 bpm, BP 134/64 mm Hg, and oxygen saturation 99% on 1 liter nasal cannula. The patient was afebrile throughout his ED course. Upon electrophysiology evaluation in the cardiac ICU, the patient was found to have a right bundle branch block and left anterior fascicular block. During admission a dual-chamber, permanent pacemaker was placed with no complications, and the patient was discharged shortly afterward.

## Discussion

While the exact sequence of events preceding the patient’s cardiac arrest is unknown, we suspect an association between the arrest and FDI. Notably, it is unclear whether the iron infusion precipitated heart block leading to cardiac arrest or if an anaphylactic-type reaction precipitated the arrest. However, an anaphylactic-type reaction seems less likely as there were no associated respiratory, cutaneous, or gastrointestinal symptoms, and the acute event resolved without epinephrine administration.

As to other etiologies of the patient’s cardiac arrest, there was no reported family history of premature coronary artery disease, dysrhythmia, cardiomyopathy, or sudden cardiac death. His most recently available ECG taken two years prior to the cardiac arrest showed a right bundle branch block and left anterior fascicular block. The patient’s family reported several episodes of syncope over the previous month. An ECG was not recorded surrounding these episodes. One episode occurred after using the bathroom, and another occurred while sitting in a chair. These prior syncopal events and the arrest may have been precipitated by complete heart block or vasovagal syncope. However, vagal etiology seems less likely as complete heart block episodes during inpatient admission showed a sinus rate in the 90s and ventricular escape in the 60s.

Laboratory results indicated that Lyme carditis, hyperkalemia, and hypothyroidism were also unlikely etiologies (Table), and the patient was not prescribed any atrioventricular blocking medications. Hypophosphatemia has been associated with parenteral iron administration, including the iron isomaltoside/FDI formulation^3^ and may result in myocardial contractility impairment and sudden cardiac death. However, lab results showed phosphorous levels within normal limits. Iron overload may exacerbate preexisting conduction disease and lead to heart block, but iron studies were within normal limits during admission, making iron overload an unlikely etiology^4^ (Table 1).

In the absence of other obvious causes, the iron infusion may have exacerbated an underlying conduction abnormality and precipitated the arrest. However, one limitation of this report is that this patient had multiple comorbidities, including prior syncopal events. While the FDI infusion was temporally associated with this patient’s cardiac arrest, we cannot conclude for certain that the infusion was the sole contributor to the event as additional factors may have played a role.

The pathophysiologic basis for FDI causing cardiac arrest is not entirely clear. Animal models have demonstrated that acute iron toxicity leads to decreased myocardial contractility and cardiac output.^5^^,^^6^ While these effects have not been previously described following parenteral iron administration, we hypothesize that FDI may have exacerbated this patient’s underlying cardiac abnormalities and precipitated the cardiac arrest. Rose and colleagues demonstrated that chronic iron overload reduces voltage dependent L type alpha 1D subunit calcium channel (Ca_V_1.3) expression in the sinoatrial node, atria, atrioventricular node, and proximal ventricular conduction system leading to bradycardia, PR-interval prolongation, heart block, and conduction deficits.^4^

While our patient did not have chronic iron overload based on lab results, it is conceivable that rapid administration of IV iron could acutely precipitate similar effects in a patient with underlying conduction abnormalities, despite the administered iron not yet having sufficient time to distribute to the tissues. Another possible pathophysiologic explanation may be Kounis syndrome, a syndrome characterized by acute coronary events including allergic coronary vasospasm, allergic myocardial infarction, or stent thrombosis secondary to a hypersensitivity reaction.^7^ Kounis syndrome has also been implicated in manifesting as fatal complete heart block.^8^ Considering parenteral iron’s history of inducing acute hypersensitivity reactions, it is plausible that parenteral iron may induce Kounis syndrome and precipitate complete heart block and cardiac arrest in a patient with underlying conduction abnormalities.

Serious adverse events caused by parenteral iron infusions are rare and typically characterized as anaphylactic-type reactions, with one study finding only two documented serious adverse events requiring epinephrine administration out of 35,737 unique iron infusions.^1^ Ferric derisomaltose has been demonstrated as safe and well tolerated compared to more commonly used parenteral iron products.^9^ One study comparing FDI to iron sucrose (IS) found sinus node dysfunction in 1/1,019 patients (0.1%) and 1/506 (0.2%) patients receiving FDI and IS, respectively, and 1/1,019 (0.1%) patients receiving FDI had cardiac arrest compared to 2/506 (0.4%) in the IS group.^10^ Another study comparing FDI to usual care (no iron or oral iron) reported bradycardia rates of 2/559 (0.4%) vs 1/568 (0.2%), complete atrioventricular block rates of 1/559 (0.2%) vs 3/568 (0.5%), and cardiac arrest rates of 6/559 (1.1%) vs 15/568 (2.6%), respectively.^11^ It is unknown whether documented cardiac effects occurred acutely following parenteral iron administration or they occurred at another time during the study period as both studies analyzed the long-term safety of FDI.

Bradycardia and heart block are rarely reported in the FDI literature and are not referenced as a warning or precaution in the package insert.^12^ However, iron dextran carries a warning to use with caution in patients with preexisting cardiovascular disease.^13^ Based on this case, it may be prudent to consider this warning for newer parenteral iron products as well.

## Conclusion

Bradycardia and heart block are rarely reported in the parenteral iron literature. Although rare, it may be reasonable to consider parenteral iron as a toxicological etiology for patients presenting with complete heart block temporally associated with a parenteral iron infusion, particularly in patients with underlying conduction abnormalities. In the case of ongoing clinical instability when other causes have been sufficiently ruled out and acute iron toxicity or chronic iron overload is suspected, consultation with the local poison control center for consideration of deferoxamine for iron chelation may be reasonable.
